# Suicide Risk Screening: A New Method to Identify At-Risk Students from General College Populations

**DOI:** 10.3390/bs16091576

**Published:** 2026-09-04

**Authors:** Qingshan Liu, Heyang Zhang, Yueqin Hu, Jie Zhang, Fang Luo

**Affiliations:** 1 Faculty of Psychology, Beijing Normal University, Beijing 100875, China; liuqingshan@mail.bnu.edu.cn (Q.L.); heyang.zhang@mail.bnu.edu.cn (H.Z.); yueqinhu@bnu.edu.cn (Y.H.); 2 Department of Sociology, SUNY Buffalo State University, Buffalo, NY 14222, USA; zhangj@buffalostate.edu

**Keywords:** suicide risk screening, university students, Composite Key Conditions (CKC), explainable AI, population-based mental health

## Abstract

University-wide suicide risk screening is essential but difficult to implement at scale because professional counselling resources are limited and conventional tools often show low predictive value in low-prevalence populations. To support transparent and clinically interpretable campus mental health workflows, this study introduces and validates the Composite Key Conditions (CKC) method, an interpretable, computationally efficient screening approach for identifying students who match counsellor-defined suicide risk categories. A multi-phase, multi-site study involving 8819 students from two universities in the development and cross-site validation phases was conducted, followed by independent external validation at a third university. In the initial wave, students completed self-report questionnaires in November 2023, after which approximately 20% were selected and invited for centralized counsellor evaluation; the assessments were conducted in December 2023. From January to June 2024, 320 students who subsequently attended psychological services (103 at University A and 217 at University B) were assessed through the cohort pathway. CKC was further evaluated in October 2025 using an independent sample of 22,205 students from a third university. The CKC method, developed from the initial phase, correctly classified all students assigned to the suicide risk category during centralized assessment (sensitivity = 1.000, specificity = 0.901). When applied across both centralized and cohort assessments (yielding 89 counsellor-defined suicide risk cases), the method maintained high performance (sensitivity = 0.888, specificity = 0.904, PPV = 0.086). In the independent validation sample, the method correctly classified 17 of the 22 students identified by the internal reporting system as meeting its high-risk criteria, with a specificity of 0.923. CKC provides an interpretable and efficient approach to suicide risk screening that is suitable for large-scale implementation in university settings. By producing transparent, auditable risk rules rather than opaque predictions, CKC is positioned to complement (not replace) counsellor judgement and to support timely triage and referral within campus mental health service systems.

## 1. Introduction

Suicide is a leading cause of death worldwide, claiming approximately 703,000 lives each year, with adolescents and young adults being particularly vulnerable (World Health Organization, 2025a, 2025b). Although the overall age-standardized suicide mortality rate in China declined substantially between 2010 and 2021, suicide mortality among individuals aged 15–24 years—an age group that substantially overlaps with the university student population—showed a marked reversal in trend, decreasing from 2010 to 2017 but increasing significantly from 2017 to 2021 (APC = 19.6%, 95% CI: 8.6–31.8%; Zhao et al., 2023). This recent trend further underscores the importance of developing feasible and effective suicide risk screening strategies for Chinese university populations. Despite decades of research, prevention remains challenging because suicidal ideation and behaviour can emerge rapidly and unpredictably. University students face intensifying academic, social, and psychological stressors and therefore represent a critical population for early screening and intervention (Wilcox et al., 2010). Yet, in higher education settings, screening must be feasible at scale and actionable within campus service constraints, and effective, context-appropriate tools remain scarce (US Preventive Services Task Force, 2022).

A key real-world challenge is that campus mental health services typically operate under limited professional capacity, which makes delayed identification and delayed intervention more likely. In this setting, screening tools are only useful if they can support a stepped-care workflow. That means they should reliably prioritize students who most need rapid follow-up, while avoiding an unmanageable false-positive burden that overwhelms counsellors and referral systems. However, existing suicide risk screening tools exhibit well-documented limitations. Many rely on direct self-report items about suicidal thoughts or behaviours, which may evoke discomfort and social desirability bias, thereby reducing response validity. Moreover, no universally accepted or standardized tool exists for suicide risk screening in educational settings. Instruments such as the Patient Health Questionnaire (PHQ-9) and the Columbia–Suicide Severity Rating Scale (C-SSRS), while clinically useful, have shown limitations in predictive power when used as stand-alone suicide risk assessment tools (Na et al., 2018; Posner et al., 2011). More broadly, commonly used risk scales have also demonstrated limited predictive accuracy and modest positive predictive value in prospective clinical evaluations (Quinlivan et al., 2018). In the context of university-wide screening, these limitations may be particularly consequential because low base rates can translate even modest false-positive rates into substantial referral burdens (McHugh & Large, 2020).

Against this backdrop, recent developments in data science and artificial intelligence have motivated the use of machine learning (ML) for suicide risk prediction. In principle, ML models can process large, multidimensional data and detect subtle associations that are difficult to encode in traditional scoring rules. In practice, however, the clinical and operational utility of many ML approaches remains controversial. As McHugh and Large noted, the “black-box” nature of many ML algorithms can undermine interpretability, limiting clinicians’ trust and hindering real-world adoption (McHugh & Large, 2020). This concern is particularly salient in campus settings where screening outputs must be communicated, justified, and used to guide concrete actions. Additionally, conventional logistic regression approaches may underestimate risk in rare event contexts, increasing the chance that individuals needing urgent support are missed (Claassen et al., 2014). More broadly, the campus deployment context introduces further constraints that are central to AI-enabled student mental health work, including cross-institution generalizability, the need for auditable decision rules, and responsible governance related to privacy and ethical boundaries.

Beyond considerations of implementation and transparency, the selection of candidate indicators for suicide risk screening should also be guided by contemporary theories of suicidal behaviour. These theories emphasize that suicidal outcomes emerge from the interaction between proximal suicide-related states and broader vulnerability factors rather than from a single indicator. For example, the Integrated Motivational–Volitional (IMV) model conceptualizes suicidal behaviour as a dynamic process involving motivational factors and factors that influence the transition from suicidal thoughts to suicidal acts (O’Connor & Kirtley, 2018). Consistent with this perspective, previous research has suggested that suicide-related risk reflects both proximal and distal factors that may contribute to heterogeneous risk profiles across individuals (Pickles et al., 2010).

To address these challenges, this study introduces and validates the Composite Key Conditions (CKC) method as an interpretable, computationally efficient, rule-based screening framework for identifying students who match the study’s counsellor-defined suicide risk category in general college populations. CKC is designed to support triage within a stepped-care workflow by providing explicit and reviewable screening conditions rather than opaque predictions. It is intended to complement counselling services by helping prioritize follow-up and referral, rather than to replace professional judgement. Machine learning methods were used during development for feature selection and benchmarking, whereas the final CKC procedure applies predefined and auditable decision rules and does not learn or adapt during deployment. We evaluate CKC using a multi-phase, multi-site design across multiple universities and benchmark its performance against common machine learning models to examine the trade-off between model complexity, interpretability, and operational feasibility in low-prevalence campus screening.

## 2. Methods

### 2.1. Participants and Procedure

This multi-phase, multi-site study was conducted between November 2023 and October 2025 among students from three public universities in Beijing, China, with the goal of evaluating an interpretable, rule-based screening approach that could be operationalized within campus mental health service workflows. Universities A and B were used for method development and cross-university validation, whereas university C served as an independent external validation sample to evaluate the portability of the screening procedure.

A total of 8819 students participated in the development and internal validation phases, including 2885 from University A and 5934 from University B. University B included both undergraduate and graduate students, whereas University A consisted solely of undergraduates. The independent validation sample comprised 22,205 students from University C. Participants ranged in age from under 18 to over 30 years (see Table 1), with gender distribution reported in Table 1.

The overall procedure followed a screening-to-triage logic consistent with stepped-care campus mental health practice. Students first completed a basic questionnaire battery designed to capture both near-term crisis indicators and longer-term vulnerability indicators. For a subset of students who were identified as potentially at risk in routine campus practice, counsellors at the Center for Psychological Counselling Service (CPCS) conducted follow-up evaluation and assigned risk levels based on professional assessment. These counsellor ratings were used as the primary criterion for evaluating screening performance because they better reflect real-world triage decisions than self-report alone. Importantly, the proposed CKC approach is intended to support prioritization and referral within campus services and to complement counsellor judgement, rather than to replace clinical decision making.

The study was embedded in routine university-wide mental health screening and counselling workflows coordinated by the Center for Psychological Counselling Service (CPCS). Data collection and risk evaluation followed a three-wave procedure aligned with stepped-care triage practices in campus settings. The study was conducted across three sequential waves spanning November 2023 to October 2025. In the first wave, participants completed the initial questionnaire assessment in November 2023, followed by counsellor interviews in December 2023. The second wave comprised the cohort assessment, which was conducted on a rolling basis from January to June 2024. The third wave, conducted in October 2025, consisted of independent external validation at University C.

#### 2.1.1. First Wave: Initial Questionnaire and Counsellor Assessment

During an annual university-wide pre-screening organized by CPCS, participants completed a demographic form followed by the basic questionnaire assessing psychological health and risk factors. University staff were permitted to add additional questionnaires according to local needs. As part of the routine clinical screening process of the university counselling centre, approximately 20% of students were selected and invited for counsellor evaluation. The proportion invited was determined based on the centre’s previous service experience and practical capacity for clinical follow-up, rather than retrospectively according to the CKC classification. Among those invited, 104 students at University A and 402 students at University B subsequently completed counsellor assessment.

Licenced clinical psychologists and social workers evaluated participants during counselling sessions and documented each encounter in the CPCS database. Participants were categorized into three risk levels based on standardized counsellor assessment criteria: no risk, indicating no suicide-related concerns or clinically significant mental distress; mental risk, indicating psychological distress without suicidal ideation or suicidal behaviour; and suicide risk, indicating the counsellor-defined suicide risk category based on suicidal ideation severity and suicide-related clinical indicators, including suicidal ideation, suicide plans, or previous suicide attempts. Because there is no universal gold standard for suicide risk classification, standardized criteria were applied uniformly across sites to enhance inter-rater consistency and reliability (Saab et al., 2022).

#### 2.1.2. Second Wave: Cohort Assessment

The second wave consisted of cohort assessments conducted on a rolling basis from January to June 2024. During this period, students who subsequently attended the Center for Psychological Counselling Service (CPCS) for psychological services were assessed according to routine service procedures. A total of 320 students participated in this cohort pathway, including 103 students from University A and 217 from University B. Counsellors conducted comprehensive assessments and assigned risk classifications using the same standardized criteria described above. Because participation in this cohort pathway was based on subsequent help-seeking and service attendance, it represented a help-seeking cohort rather than a fixed-time follow-up assessment of the original screened population.

#### 2.1.3. Third Wave: Independent External Validation

The third wave was conducted in October 2025 at University C and served as an independent external validation of CKC. A total of 22,205 students participated. Unlike Universities A and B, University C used an internal reporting system rather than counsellor-based risk classification as the reference criterion. CKC was applied using the fixed operating cutoff established in the development and cross-site validation phases, without recalibration. This wave was used to evaluate the portability of CKC under a different operational and assessment context.

### 2.2. Measures

To support interpretable, rule-based screening, nine validated instruments comprising 45 items were used to capture both explicit and implicit indicators of suicide risk as operationalized in the CKC framework (see Appendix A and Appendix B). Measures were selected to be feasible for large-scale administration while providing transparent constructs that can be reviewed by stakeholders in a campus mental health management context.

Explicit indicators reflected near-term risk and included suicidal ideation assessed via the HDSQ suicidal subscale, personal and familial psychiatric history (e.g., parental depression or suicide), and history of suicide attempts, psychotropic medication use, or major health problems. Implicit indicators reflected more chronic psychological vulnerability and included depressive symptoms (SCL-90 depression subscale), childhood trauma (CTQ), low self-esteem (RSE), defeat and entrapment (SDES), psychological strain (PSS), and psychological pain (MBPPAS). Childhood trauma has been associated with later suicide-related vulnerability and may operate through mechanisms including impulsivity and executive functioning (Rogerson et al., 2022). In the CKC workflow, explicit indicators are designed to flag potential acute crisis needs, whereas implicit indicators help characterize longer-term vulnerability that may warrant monitoring or preventive support in a stepped-care system. This integration is consistent with contemporary multifactorial models of suicide risk, which conceptualize suicidal thoughts and behaviours as emerging from interactions among multiple psychological processes rather than a single symptom domain (O’Connor & Kirtley, 2018; Ballard & Zarate, 2018). From this perspective, explicit indicators in CKC capture more proximal crisis-related signals, whereas implicit indicators characterize enduring psychological vulnerabilities that may contribute to suicide-related risk over time. Psychological pain has been proposed as a central component of suicidal processes (Shneidman, 1993), and empirical evidence has further demonstrated its association with suicidality beyond traditional affective symptoms (Troister & Holden, 2010). Moreover, individual differences in the tolerance of psychological pain may influence how underlying vulnerability is translated into suicidal thoughts or behaviours (Meerwijk & Weiss, 2018). Therefore, the integration of near-term explicit indicators and longer-term implicit indicators allows CKC to capture complementary dimensions of suicide-related risk.

All self-report instruments were administered as part of the basic questionnaire (and any locally added instruments where applicable). Measures assessed both near-term explicit risk indicators and longer-term implicit vulnerability factors used in subsequent CKC computation. All items were rated on Likert-type scales (0–4 unless otherwise noted), and mean subscale scores were computed before integration into the CKC equation. Reliability coefficients (Cronbach’s α) for the continuous variables ranged from 0.81 to 0.93, indicating excellent internal consistency.

### 2.3. Statistical Analysis

#### 2.3.1. Data Analysis Overview

Participant characteristics were summarized using descriptive statistics. The primary objective was to evaluate CKC as a screening method for classifying students according to the study’s counsellor-defined suicide risk category in a low-prevalence campus population, where screening utility depends not only on classification performance but also on operational feasibility under limited counselling capacity. Accordingly, screening performance was assessed using sensitivity, specificity, and positive predictive value (PPV).

University A was used for CKC development and preliminary cutoff calibration, whereas University B was used for cross-site validation and further evaluation of cutoff stability. After cross-site evaluation, the final operating cutoff was fixed and subsequently applied without recalibration to University C for independent validation. Model tuning focused on selecting an operating cutoff that optimizes discrimination while remaining feasible for campus service capacity. The cutoff parameter was calibrated through cross-validation using Youden’s index (Youden, 1950). After selection, the cutoff was fixed and applied consistently across datasets to assess stability and generalizability. University C served as an independent validation sample to evaluate performance without recalibration.

#### 2.3.2. Statistical Assessment

After completion of the basic questionnaire, staff at the Center for Psychological Counselling Service (CPCS) conducted follow-up assessments for students referred through routine campus screening and counselling pathways. Counsellors assigned each assessed student to one of three predefined categories based on standardized assessment criteria. These counsellor-defined categories served as the reference classification for evaluating CKC because they reflected routine stepped-care triage decisions more closely than questionnaire responses alone. However, because counsellor assessment was conducted only for a selected subset of students, these classifications should not be interpreted as a gold standard for the entire screened population.

Across Universities A and B, 506 students received counsellor assessment during the centralized assessment pathway, including 104 students from University A and 402 from University B. Risk categories were coded as follows: 0 = no risk, 1 = mental risk, and 2 = suicide risk. The definitions of these categories are provided in Section 2.1.1.

**Figure 1:**
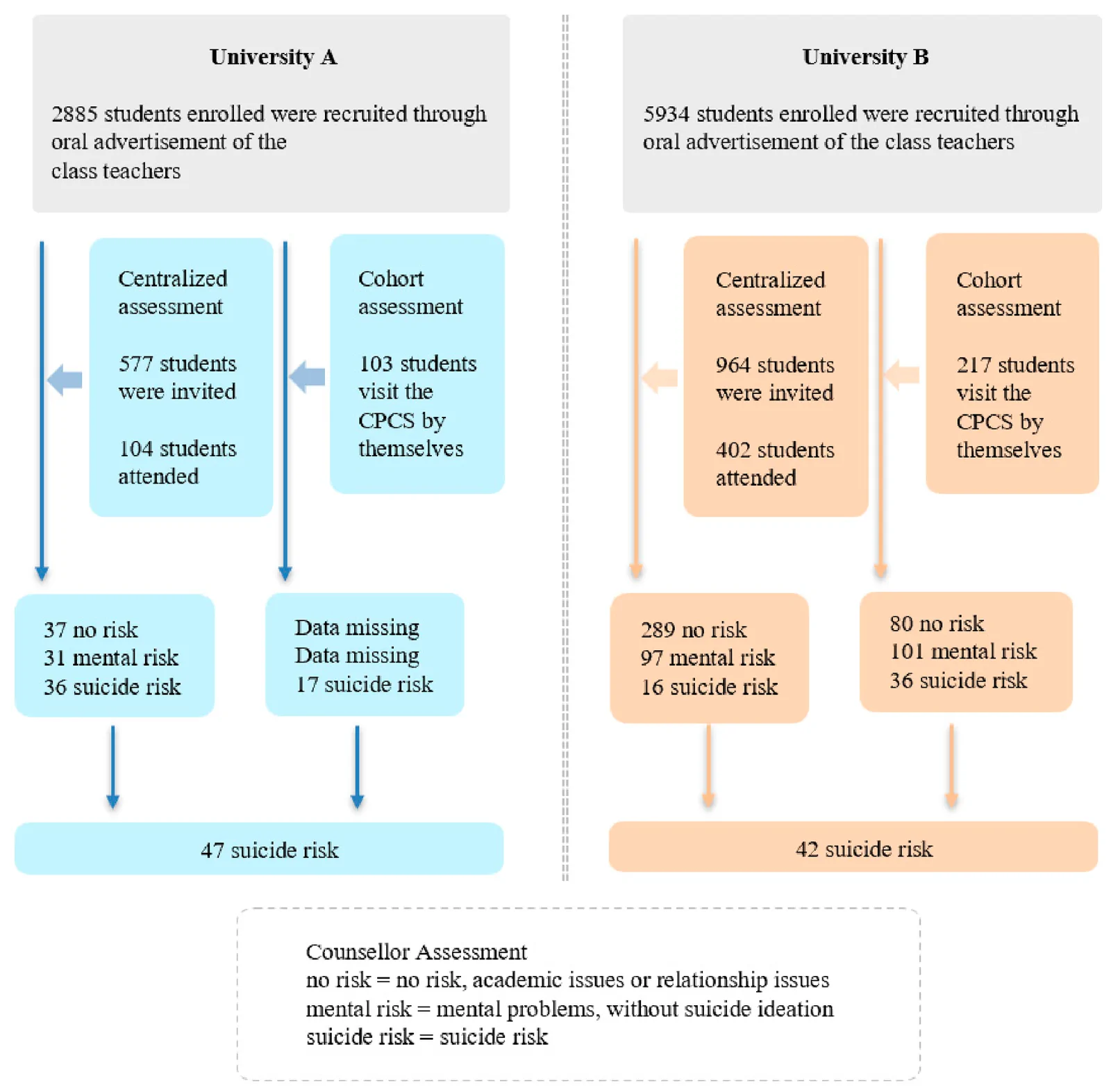
Participant flow across screening and assessment pathways.

At University A, 104 students completed centralized counsellor assessment, of whom 37 (35.6%) were classified as no risk, 31 (29.8%) as mental risk, and 36 (34.6%) as suicide risk category. The cohort assessment pathway subsequently identified 17 suicide risk classifications, six of whom overlapped with suicide risk cases identified through the centralized assessment pathway. After accounting for this overlap, 47 unique students were included in the suicide risk category. At University B, 402 students completed centralized counsellor assessment, of whom 289 (71.9%) were classified as no risk, 97 (24.1%) as mental risk, and 16 (4.0%) as suicide risk category. The cohort assessment pathway subsequently identified 36 suicide risk classifications, 10 of whom overlapped with suicide risk cases identified through the centralized assessment pathway. After accounting for this overlap, 42 unique students were included in the suicide risk category. The participant flow, overlap across assessment pathways, and deduplication process are summarized in Figure 1. For performance evaluation, students assigned to the counsellor-defined suicide risk category were treated as the positive reference class.

#### 2.3.3. Machine Learning Benchmarking and Development Support

During the developing stage, several conventional machine learning algorithms were evaluated as benchmark methods in this low-prevalence screening context (McHugh & Large, 2020). The models included logistic regression, random forest, decision tree, support vector machine (SVM), k-nearest neighbours (KNN), Bayesian classifier, and multilayer perceptron (MLP). Each model was trained using both the original dataset and a dataset balanced using the Synthetic Minority Oversampling Technique (SMOTE) (Thabtah et al., 2020).

These machine learning analyses served two purposes: first, to provide benchmark performance against which the CKC screening procedure could be compared; and second, to support exploratory feature selection during method development. They were not part of the final deployed CKC procedure. The final CKC method applies predefined scoring rules, explicit logical conditions, and a fixed operating cutoff, and it does not update its parameters or learn from new observations during use.

Although some benchmark models achieved high sensitivity in the training data, cross-university evaluation showed unstable performance and substantial false-positive burden. For example, when University A data were used for training and University B data for testing, some SMOTE-based models achieved very high sensitivity but low positive predictive value. Reversing the training and testing universities produced similarly unstable results.

Performance metrics for the benchmark models, including sensitivity, specificity, positive predictive value (PPV), negative predictive value (NPV), and Youden’s index, are reported in Appendix C, Table A9. Youden’s index was calculated as sensitivity + specificity − 1. In low-prevalence settings, modest PPV can result in a large number of false-positive classifications, which may impose an impractical burden on campus counselling services. These findings motivated the development of a more transparent and operationally feasible rule-based screening procedure.

### 2.4. Composite Key Conditions (CKC) Method

#### 2.4.1. Conceptual Framework

To address the limited interpretability and operational instability of complex machine learning models in large-scale campus screening, we developed the Composite Key Conditions (CKC) method as a transparent, rule-based screening approach. CKC was designed to combine clinically interpretable indicators through predefined scoring and logical conditions, thereby supporting reviewable and computationally efficient screening decisions.

**Figure 2:**
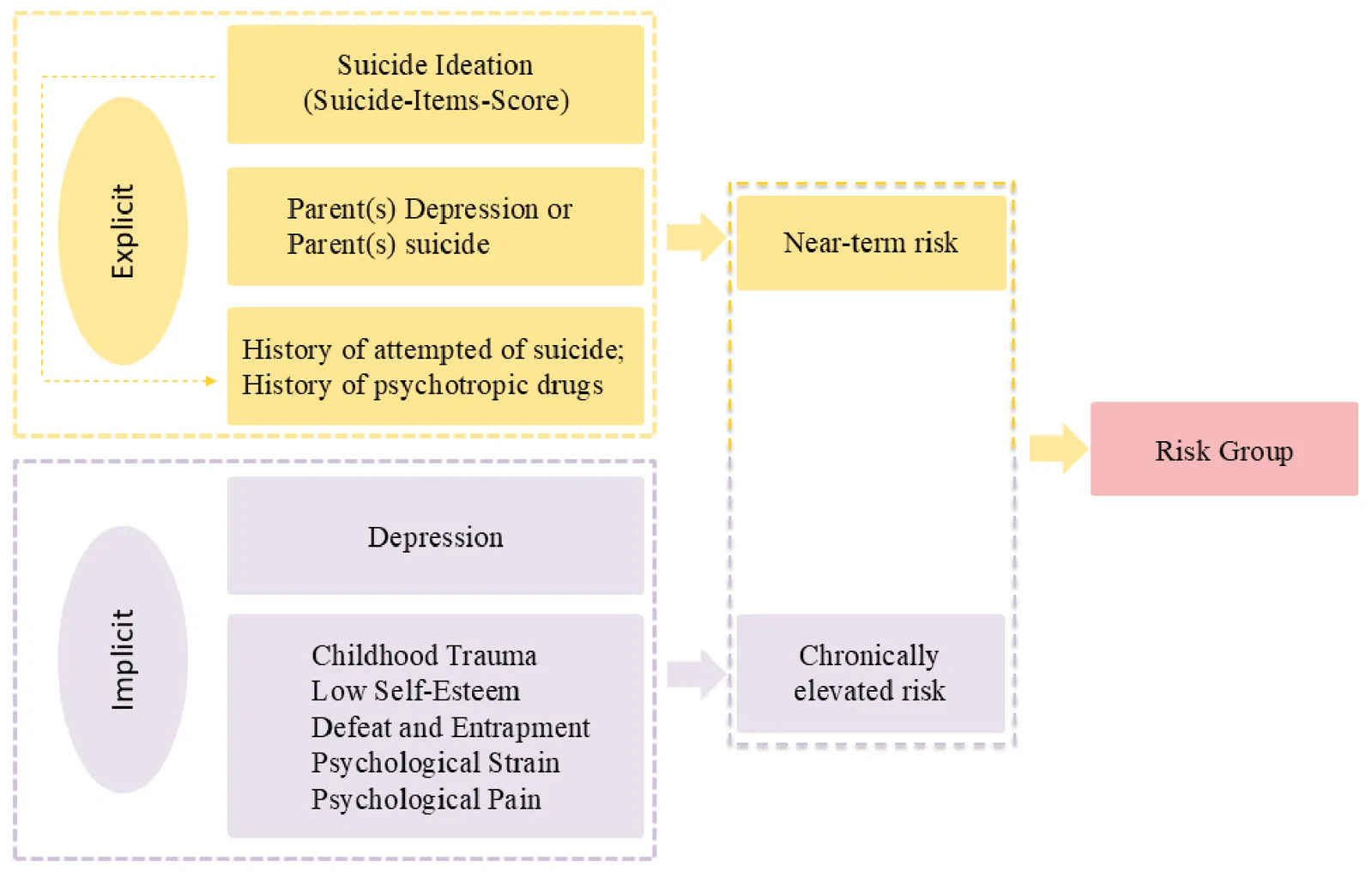
Model flow chart.

The framework integrates explicit and implicit psychological indicators (see Figure 2) that represent complementary components of the screening process. Explicit indicators capture more immediate suicide-related signals, including suicidal ideation, personal and familial psychiatric history, previous suicide attempts, psychotropic medication use, and major health problems. Implicit indicators capture broader and potentially more enduring psychological vulnerabilities, including depressive symptoms, childhood trauma, low self-esteem, defeat and entrapment, psychological strain, and psychological pain.

This distinction does not imply that CKC predicts the future occurrence of suicidal behaviour. Rather, it provides a structured way to combine near-term suicide-related indicators with longer-term vulnerability indicators when classifying students according to the study’s operational screening criteria. This multifactorial structure is consistent with recommendations that suicide screening should not rely on a single symptom dimension or scale alone (Bryan et al., 2022; Saab et al., 2022).

CKC is intended for use within screening-to-triage workflows. Its outputs consist of explicit and auditable screening conditions that can be reviewed and communicated by campus mental health professionals. The procedure is intended to support prioritization of follow-up and referral within a stepped-care framework, rather than to replace professional assessment or to provide a definitive prediction of future suicidal behaviour.

#### 2.4.2. Algorithm and Computation

CKC first computes a Total Mean Score (*TMS*) representing the combined level of the continuous psychological indicators included in the screening procedure. Each continuous scale score is divided by the number of retained items from that scale, and the resulting mean scores are summed to obtain the *TMS*:

$$TMS=\frac{L-RSE}{items(L-RSE)}+\frac{DES-S}{items(DES-S)}+\frac{PSS}{items(PSS)}+\frac{MBPPAS}{items(MBPPAS)}+\frac{SCL-Depression}{items(SCL-Depression)}+\frac{CTQ}{items(CTQ)}$$

In this equation, L-RSE denotes low Rosenberg Self-Esteem, DES-S denotes the short version of the Defeat and Entrapment Scale, PSS denotes the Psychological Strain Scale, MBPPAS denotes the Mee–Bunney Psychological Pain Assessment Scale, SCL-depression denotes the SCL-90 depression subscale, and CTQ denotes the Childhood Trauma Questionnaire.

The CKC procedure also incorporates dichotomous indicators, including parental depression history, parental suicide history, previous suicide-attempted history, and psychotropic drug history. These variables are treated as predefined screening indicators rather than as independent predictions of future suicidal behaviour.

A participant was flagged by CKC when at least one of the predefined key conditions was met. The key conditions were: (1) TMS≥12; (2) a suicidal ideation score of ≥5, or a suicidal ideation score of 4 together with an SCL-90 depression score of ≥2; and (3) the presence of at least one predefined dichotomous indicator.

CKC then combines the continuous and dichotomous components to calculate an overall screening score:

$$Risk\;Score=TMS+\beta \times PD+\beta \times HPS+\beta \times HAS+\beta \times HPD+\beta \times \left(SuicideItemRisk\right)+\beta \times (SuicideDepression)$$

In this study, the term *Risk Score* refers to the numerical screening score generated through the CKC procedure. It should not be interpreted as an estimated probability of future suicidal behaviour. A participant was classified as CKC-positive when the calculated *Risk Score* met or exceeded the prespecified cutoff value, *β*. The cutoff was calibrated through cross-validation using Youden’s index (Youden, 1950) and was subsequently fixed for cross-site and external evaluation. Sensitivity analyses were conducted by evaluating CKC performance across alternative cutoff values to examine the robustness of the selected operating threshold.

### 2.5. Ethical and Safety Protocol

The study was conducted in accordance with the Declaration of Helsinki (Saab et al., 2022; US Preventive Services Task Force, 2022) and was approved by the relevant institutional ethics committees. All participants provided electronic informed consent before data collection and were informed that participation in the questionnaire, counsellor assessment, and any subsequent follow-up was voluntary. Participants younger than 18 years were 17-year-old university students who had not yet reached their 18th birthday at the time of data collection. Their inclusion and use of the same electronic informed-consent procedure as other participating university students were covered by the protocol approved by the ethics committee. Participants could decline or withdraw from any component without penalty. All research data were anonymized and managed in accordance with institutional privacy and data protection procedures.

Students whose questionnaire responses or counsellor assessments indicated an immediate need for further attention were referred to the relevant on-campus counselling service for face-to-face reassessment. Qualified mental health professionals determined the appropriate level of response according to the student’s current condition. When clinically indicated, crisis management procedures included immediate crisis intervention, development of an individualized safety plan, identification of personal coping strategies and available social support resources, documentation of emergency contact information, and scheduled follow-up.

For students requiring a higher level of care, counsellors could initiate psychiatric referral and, when necessary and permitted under institutional protocols, contact family members or other designated support persons. Students requiring continued support were monitored through follow-up counselling appointments and reassessment. The CKC classification was used only to support screening and prioritization; all decisions concerning crisis intervention, family or emergency contact, psychiatric referral, and ongoing management were made by qualified mental health professionals in accordance with institutional procedures.

## 3. Results

### 3.1. Preliminary Model

Sensitivity and specificity were used as primary indicators of screening performance in a low-prevalence campus setting. In the present study, sensitivity represents the proportion of students in the counsellor-defined suicide risk reference category who were classified as CKC-positive, whereas specificity represents the proportion of students outside that reference category who were classified as CKC-negative (Horowitz et al., 2012). Sensitivity and specificity were calculated across candidate cutoff values to identify an operating point that balanced classification performance with the false-positive burden imposed on campus counselling services.

At a cutoff score of 14, all students assigned to the suicide risk category in the reference assessment were classified as CKC-positive (sensitivity = 1.00). Specificity at University A was 0.890, indicating that most students outside the suicide risk reference category were classified as CKC-negative. For context, Na et al. used PHQ-9 items to classify suicidal ideation and reported a sensitivity of 0.466 (0.420–0.513) and a specificity of 0.650 (0.602–0.695) (Na et al., 2018). Choi et al. evaluated the Suicide Screening Questionnaire-Observer Rating (SSQ-OR) and reported a sensitivity of 0.73 and a specificity of 0.792 at the optimal cutoff (Choi et al., 2022). In this preliminary analysis, CKC showed higher sensitivity and comparable or higher specificity relative to these reported values; however, these comparisons should be interpreted cautiously because the studies used different samples, reference criteria, and assessment procedures.

Lower cutoff values increased the proportion of students classified as CKC-positive but also increased the number of false-positive classifications relative to the counsellor-defined reference category. Based on this trade-off, *β* = 14 was retained as the preliminary operating cutoff during the initial item reduction stage. After subsequent cutoff calibration and sensitivity analyses, the final operating threshold was determined as *β* = 12 for cross-site evaluation and validation.

### 3.2. Final Model

The original questionnaire included 45 risk-related items, which was considered excessive and potentially fatiguing for students in large-scale screening. To streamline assessment while maintaining screening performance, we applied an integrative feature reduction approach combining multiple linear regression, decision tree analysis, and machine learning based feature selection. The decision tree model was constrained to a maximum depth of four, using counsellor-defined risk classification as the reference outcome. Variables with higher information gain and entropy-based importance were retained. Feature retention was determined based on the combined evidence from statistical significance, decision tree contribution, and machine learning feature importance, while maintaining clinical interpretability of the retained indicators. All feature selection procedures were conducted within the development dataset to reduce the risk of overfitting before external validation. Through this process, 15 statistically significant items were identified via regression analysis, and a final set of 29 items was selected from the original 45-item pool (see Appendix B).

Using the refined item set, sensitivity, specificity, and Youden’s index were recalculated across candidate thresholds. A cutoff score of 12 provided a favourable balance, with sensitivity reaching 1.000 and specificity reaching 0.876, while maintaining strong overall screening utility. Although nearby thresholds (11–12) showed comparable performance, *β* = 12 was selected as the final operating threshold for subsequent cross-site validation.

### 3.3. Cross-Sectional Validation

The CKC method was developed using University A data and validated using University B data, where 5934 students completed the questionnaire. At University B, 16 students were assigned to the suicide risk category and 97 to the mental risk category. Despite differences in pre-screening procedures and demographic composition across campuses, CKC maintained stable screening performance when transported across sites. Consistent with the intended screening-to-triage use case, cutoff selection was evaluated not only for discrimination but also for the operational burden imposed by false-positives.

As detailed in Table 2, the sensitivity–specificity trade-off was evaluated across multiple candidate thresholds in the cross-site validation sample (University B). To align the expected screening-positive proportion with the Center for Psychological Counselling Services (CPCS) triage capacity (approximately 20% of students could be selected and invited for counsellor evaluation), a cutoff in the 11–12 range was selected as the primary operating window, balancing sensitivity with an operationally manageable false-positive burden. Subsequent cross-campus evaluation supported retaining the selected operating cutoff (see Table 3).

### 3.4. Cohort Assessment

To evaluate CKC performance in the cohort assessment pathway, cohort assessments were conducted on a rolling basis from January to June 2024 among students who subsequently attended psychological services at the CPCS. A total of 217 students from University B participated in this cohort pathway. Counsellors documented 80 no risk, 101 mental risk, and 36 suicide risk classifications.

Of the 36 suicide risk cases identified through the cohort assessment pathway, 10 overlapped with suicide risk cases identified through the centralized assessment pathway. After accounting for this overlap, a total of 42 unique students out of the 5934 participants at University B were included in the counsellor-defined suicide risk category across the centralized and cohort assessment pathways.

**Figure A1:**
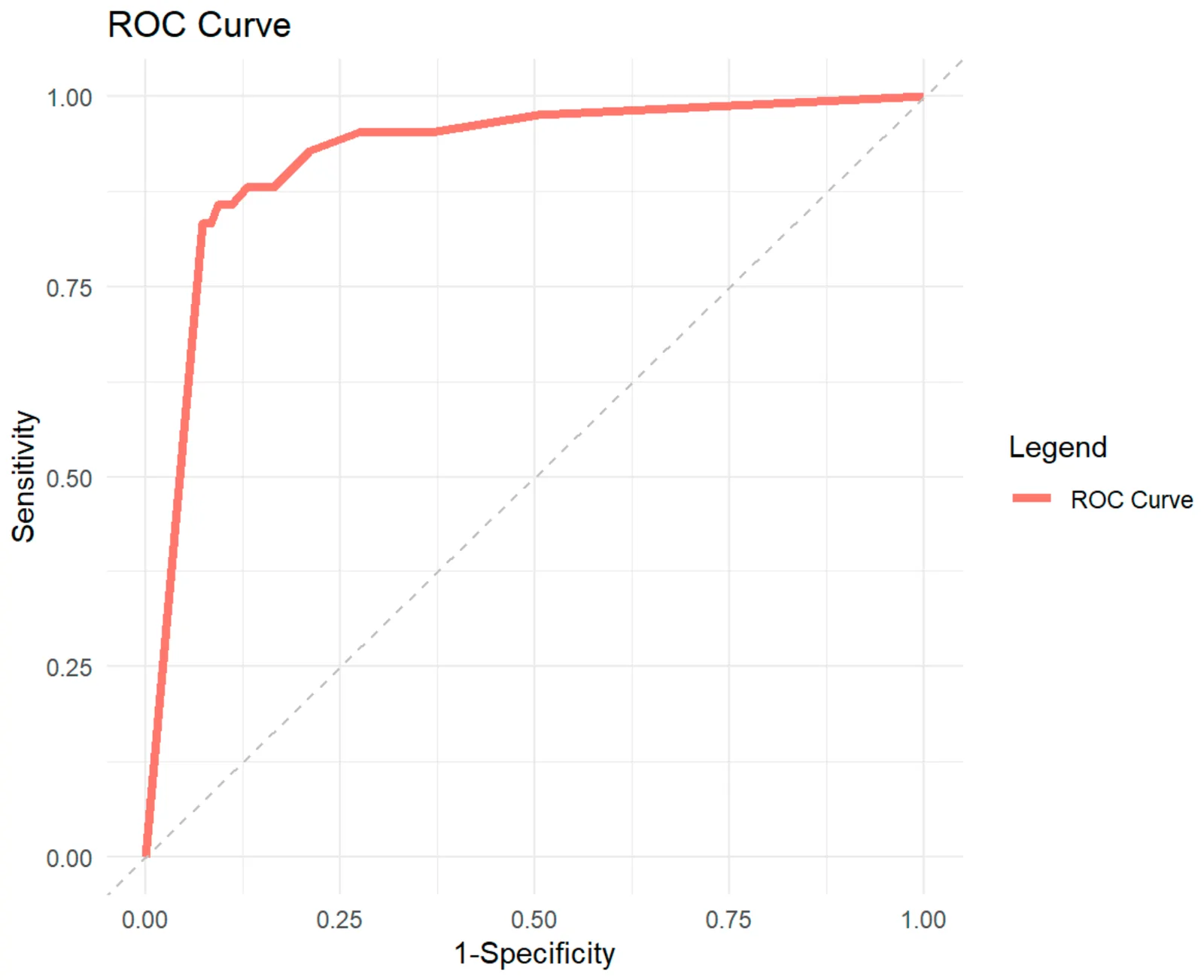
Receiver operating characteristic curve analysis for the suicide screening questionnaire—assessment rating. The dashed diagonal line represents chance-level performance (AUC = 0.5).

CKC achieved a sensitivity of 0.833, specificity of 0.916, and PPV of 0.066 (see Table A10 and Figure A1 in Appendix C). Receiver operating characteristic (ROC) analysis yielded an area under the curve (AUC) of 0.885. Of the seven cases initially not classified as CKC-positive, four were subsequently reassigned outside the suicide risk category, leaving three students in the final counsellor-defined suicide risk category who remained CKC-negative.

### 3.5. Large-Scale Traceability Validation

To examine generalizability under a different operational environment, CKC was further evaluated at University C with 22,205 participants. Unlike Universities A and B, university C relied on an internal reporting system rather than counsellor evaluations. Among 22 students identified by the internal reporting system as meeting its high-risk criteria, CKC correctly classified 17 as CKC-positive, with a specificity of 0.923. This large-scale independent evaluation provides additional evidence regarding the portability of CKC in educational settings.

### 3.6. Overall CKC Performance

Table 3 provides a cross-campus summary of CKC operating characteristics across cutoff scores. Using cutoff = 12 as the primary operating point, CKC showed consistent performance in the counselling-anchored samples (University A sensitivity = 0.936, specificity = 0.879; University B sensitivity = 0.833, specificity = 0.916) and remained robust in the large-scale validation sample (University C sensitivity = 0.773, specificity = 0.923). When data from Universities A and B were combined (89 counsellor-defined suicide risk cases in total), the CKC method achieved an overall sensitivity of 0.888, specificity of 0.904, and PPV of 0.086 at the same cutoff. Although PPV was modest—consistent with the low prevalence of the counsellor-defined suicide risk category in the general student population—this operating point maintained a practical balance between sensitivity and a manageable false-positive burden for stepped-care triage in campus counselling settings.

## 4. Discussion

Accurately predicting suicide risk in general student populations remains challenging. Even well-validated instruments such as the Columbia–Suicide Severity Rating Scale (C-SSRS) show limited predictive validity when base rates are low (McHugh & Large, 2020; Saab et al., 2022). To address this gap, we developed and validated the Composite Key Conditions (CKC) method—a theory-guided and empirically optimized framework intended to balance screening performance, interpretability, and practical deployment in large-scale university screening.

A central contribution of CKC is its multi-conditional logic for integrating heterogeneous suicide-related indicators. Rather than relying on a single symptom dimension, CKC integrates near-term indicators with more enduring vulnerabilities, reflecting a dynamic systems perspective on suicidal behaviour. The inclusion of depressive symptoms, self-esteem, and prior suicide attempt history is consistent with evidence identifying these factors as key proximal and distal correlates of suicide-related outcomes (Harris & Barraclough, 1997; Probert-Lindström et al., 2020; Na et al., 2018; Paris, 2021).

Direct assessment of suicidal ideation, suicide plans, impulses, and previous suicidal behaviour remains an essential component of suicide risk screening. However, CKC was not designed merely to reproduce a simple direct-indicator rule. Its rationale is to integrate these proximal suicide-related indicators with broader psychological vulnerabilities and adverse experiences that may contribute to heterogeneous risk profiles. Accordingly, CKC is intended to complement direct suicide-related assessment by providing a multidimensional screening framework rather than replacing direct questioning.

Methodologically, our results also support using a granular assessment of suicidal ideation as one component of screening in non-clinical contexts. Consistent with later applications of brief ideation screens such as the ASQ (Horowitz et al., 2012), ideation-focused assessment can capture clinically relevant suicide-related signals while potentially reducing non-disclosure and reactivity that may occur when asking direct questions about suicidal behaviour in broad screening settings (Runeson et al., 2017). By pairing ideation severity with psychological vulnerability markers, CKC produces an interpretable screening composite for identifying students who meet the study’s operational screening criteria that is both sensitive and ethically deployable.

Across three participating university samples, CKC showed stable operating characteristics, providing preliminary evidence of portability within the sampled university settings. At the primary operating cutoff of 12, performance was strong in the counselling-anchored samples (University A sensitivity = 0.936, specificity = 0.879; University B sensitivity = 0.833, specificity = 0.916) and remained robust in the large-scale validation sample (University C sensitivity = 0.773, specificity = 0.923). When Universities A and B were combined (89 counsellor-defined suicide risk cases), the overall sensitivity and specificity were 0.888 and 0.904, respectively, with PPV = 0.086.

Importantly, the modest PPV should be interpreted in light of the low prevalence of the counsellor-defined suicide risk category in general student populations. Under such conditions, no screening tool can achieve high precision without sacrificing sensitivity (Harris & Barraclough, 1997; Tjoa & Guan, 2020). By prioritizing sensitivity while maintaining specificity around or above 0.90, CKC provides a pragmatic balance between minimizing classifications missed relative to the reference category and avoiding excessive false-positives that could overwhelm campus services. This trade-off also underscores the value of repeated assessment and stepped-care triage following an initial positive screen.

Although CKC is designed to support stepped-care triage by identifying students who meet the study’s counsellor-defined suicide risk criteria, we acknowledge that triage decisions should not rely exclusively on risk indicators or prediction-oriented frameworks. Contemporary approaches emphasize that assessment of protective factors, reasons for living, and resilience-related resources can provide complementary information for determining urgency and intervention needs. Therefore, future implementations of CKC could incorporate protective-factor assessments alongside risk screening to support more comprehensive and individualized triage decisions.

Beyond screening performance, CKC has practical advantages for implementation. Its transparent, rule-based structure allows stakeholders to understand which conditions contributed to risk classification, supporting clinician trust and facilitating communication in prevention workflows. The framework can be integrated into digital screening systems with minimal computational burden, aligning with current calls for interpretable and ethically deployable decision support methods in mental health.

From an implementation perspective, the feasibility of CKC depends not only on screening accuracy but also on respondent burden and the capacity of campus mental health services. Completion of the questionnaire battery used in routine screening generally required approximately 30 min. The reduction of the original 45-item pool to the final 29-item set was intended to further reduce respondent burden while preserving screening performance. In routine practice, approximately 20% of students were selected and invited for counsellor evaluation based on the counselling centre’s previous service experience and practical follow-up capacity. The interval between screening and counsellor assessment varied according to service availability: some students were assessed as early as the following day or within the following week, whereas the longest waiting time was approximately one month. Students requiring further attention were referred for face-to-face reassessment, with crisis intervention, individualized safety planning, psychiatric referral, and follow-up arranged when clinically indicated.

Nevertheless, generalizability should be considered cautiously. All participating institutions were located in Beijing, and cultural and educational contexts may influence symptom expression, stigma, and help-seeking. Replication across regions and subgroups will be important to evaluate transferability, calibration, and fairness.

### Limitations and Future Directions

This study’s limitations include sampling from a single metropolitan region, which may constrain generalizability. Although the inclusion of multiple universities within Beijing provides evidence of portability across institutional settings, the observed patterns may also reflect local cultural, educational, and help-seeking contexts. Another limitation concerns the incomplete verification of the reference classification. Because counsellor assessments were conducted only for selected participants rather than the entire screened population, the present study cannot determine whether some students who were not assessed after screening may have met the counsellor-defined suicide risk category. Therefore, sensitivity and specificity estimates should be interpreted as performance relative to the available reference assessments rather than complete verification of all screened students. Future studies incorporating independent clinical interviews among randomly sampled screen-negative students would provide stronger estimates of potential false-negative cases. Because participation in the cohort assessment was voluntary and based on subsequent help-seeking behaviour, students who attended psychological services during the January–June 2024 cohort period may differ from those who did not participate. This may introduce potential selection bias and should be considered when interpreting the cohort findings.

Although University C provided an independent large-scale evaluation, its reference criterion differed from the counsellor-based assessments used in Universities A and B. In addition, the demographic composition of University C differed from that of Universities A and B, particularly with respect to age and gender distributions. These differences in both reference criteria and sample composition may have contributed to variation in screening performance across sites and should therefore be considered when interpreting the portability of CKC across university populations. Future studies using harmonized reference standards and more demographically comparable samples across institutions will be important to further evaluate the generalizability of CKC. Additional limitations concern measurement characteristics and clinical interpretation. Most psychological indicators incorporated into CKC were derived from self-reported measures, which may be influenced by social desirability bias or underreporting of sensitive experiences. Although counsellor assessments followed standardized procedures, clinical judgement may still introduce potential variability across assessors. Furthermore, the current study evaluated CKC against counsellor-defined risk classifications rather than long-term clinical outcomes; future prospective studies linking CKC classifications with clinically meaningful outcomes are needed. As a screening framework, CKC also requires careful consideration of both false-positive and false-negative classifications, including unnecessary intervention burden or stigma among false-positive cases and missed opportunities for timely support among students requiring assistance.

Although risk factors were selected based on theoretical and empirical relevance, their optimal combination and weighting may vary across populations. The analytic design prioritized interpretability over algorithmic complexity, enhancing usability but potentially overlooking subtle nonlinear relations. Future work should validate CKC across more diverse regions, conduct subgroup fairness analyses across demographic characteristics, and test deployment within real-time counselling workflows to examine downstream effects on help-seeking, referral efficiency, and crisis reduction. Future longitudinal studies should examine how CKC classifications relate to subsequent help-seeking, clinical needs, and service utilization, thereby providing further evidence for its practical utility in campus mental health systems. Although the proportion of students invited for counsellor assessment was determined according to the counselling centre’s previous service experience and practical capacity, counsellor workload was not formally quantified using standardized implementation metrics such as staff hours, caseload per counsellor, or resource use. Future implementation studies should therefore prospectively evaluate staffing requirements, workload, waiting times, referral completion, and downstream service utilization to provide a more comprehensive assessment of implementation feasibility.

## Figures and Tables

**Table 1:** Demographic characteristics of the study sample.

Variable		University A	University B	University C
Gender	Participants	2885	5934	22,205
	Male	826	2002	15,210
	Female	2057	3932	6995
	Not reported	2	0	0
Age	Under 18 years	111	134	5282
	18–21	2768	2203	6349
	22–29	6	3277	9839
	30+	0	320	735
Education level	Undergraduate	2885	2166	12,655
	Graduate	0	3768	9370

**Table 2:** Sensitivity and specificity of the CKC method for classifying students according to the counsellor-defined suicide risk category at University B.

	0 (No Suicide Risk)	1 (Suicide Risk)	Sensitivity	Specificity	Youden	PPV	NPV
Count	5918	16					
Risk Score ≥ 8	995	16	1	0.8319	0.8319	0.0158	1
Risk Score ≥ 9	789	16	1	0.8667	0.8667	0.0199	1
Risk Score ≥ 10	671	16	1	0.8866	0.8866	0.0233	1
Risk Score ≥ 11	564	16	1	0.9047	0.9047	0.0276	1
Risk Score ≥ 12	513	16	1	0.9133	0.9133	0.0302	1
Risk Score ≥ 13	491	16	1	0.9170	0.9170	0.0316	1
Risk Score ≥ 14	476	16	1	0.9196	0.9196	0.0325	1
Risk Score ≥ 15	466	16	1	0.9213	0.9213	0.0332	1

**Table 3:** Performance of the Composite Key Conditions (CKC) method for classifying students according to the counsellor-defined suicide risk category.

	University A					University B			University C		
	No Risk	At Risk	Sensitivity	Specificity	Youden	Sensitivity	Specificity	Youden	Sensitivity	Specificity	Youden
Cutoff	2838	47									
Risk ≥ 4	1582	45	0.957	0.443	0.4	0.976	0.496	0.472	0.909	0.347	0.256
Risk ≥ 5	1245	45	0.957	0.561	0.519	0.952	0.634	0.587	0.864	0.5	0.364
Risk ≥ 6	987	44	0.936	0.652	0.588	0.952	0.727	0.679	0.864	0.633	0.496
Risk ≥ 7	786	44	0.936	0.723	0.659	0.929	0.79	0.719	0.864	0.731	0.594
Risk ≥ 8	635	44	0.936	0.776	0.712	0.881	0.835	0.716	0.864	0.804	0.668
Risk ≥ 9	512	44	0.936	0.82	0.756	0.881	0.87	0.751	0.818	0.856	0.674
Risk ≥ 10	439	44	0.936	0.845	0.782	0.857	0.89	0.747	0.818	0.889	0.707
Risk ≥ 11	375	44	0.936	0.868	0.804	0.857	0.908	0.765	0.818	0.91	0.728
Risk ≥ 12	344	44	0.936	0.879	0.815	0.833	0.916	0.75	0.773	0.923	0.696
Risk ≥ 13	312	43	0.915	0.89	0.805	0.833	0.92	0.753	0.773	0.929	0.702
Risk ≥ 14	305	43	0.915	0.893	0.807	0.833	0.922	0.756	0.773	0.931	0.704
Risk ≥ 15	297	43	0.915	0.895	0.81	0.833	0.924	0.758	0.773	0.932	0.705
Risk ≥ 16	296	43	0.915	0.896	0.811	0.833	0.925	0.758	0.773	0.932	0.705
Risk ≥ 17	291	43	0.915	0.898	0.812	0.833	0.926	0.76	0.773	0.932	0.705
Risk ≥ 18	290	43	0.915	0.898	0.813	0.833	0.927	0.76	0.773	0.933	0.705
Risk ≥ 19	290	43	0.915	0.898	0.813	0.833	0.927	0.76	0.773	0.933	0.705

*Note.* Table 3 summarizes CKC performance across Universities A–C at different cutoff scores. Values of 0 and 1 denote participants classified as not at risk and at risk, respectively. Sensitivity, specificity, and Youden’s index are reported for each cutoff to evaluate the robustness of the selected cutoff threshold. For University A, counts of screened not-at-risk and at-risk cases are also shown.

## Data Availability

The data presented in this study are available on request from the corresponding author due to privacy and ethical restrictions.

## Appendix A. Psychological and Clinical Indicators in the CKC Model

The Composite Key Conditions (CKC) model operationalizes suicide risk through a set of validated psychological and clinical instruments. It integrates nine measurement sources representing both explicit (near-term) and implicit (chronic) risk factors. All scales were previously validated and underwent linguistic and cultural adaptation for Chinese university students. Detailed item-level content and response formats are provided in Appendix B.

### Appendix A.1. Hopelessness Depression Symptom Questionnaire (HDSQ)

The suicidality subscale of the HDSQ is a brief measure capturing four suicidal beliefs: (a) suicidal thoughts, (b) formulation of a plan, (c) perceived control over these thoughts, and (d) suicidal impulses. It consists of four self-report items rated on a four-point Likert scale (0 = Never to 3 = Always). Metalsky and Joiner (1997) reported subscale Cronbach’s *α* = 0.70–0.86 and total *α* = 0.93.

### Appendix A.2. Rosenberg Self-Esteem Scale (RSE)

The RSE is one of the most widely used self-esteem measures, with extensive psychometric validation (Rosenberg, 1965; Gray-Little et al., 1997). It contains 10 items scored on a five-point Likert scale (0 = Strongly Disagree to 4 = Strongly Agree), adapted from the original four-point format for scale consistency. Reliability is acceptable (α ≈ 0.77), and the coefficient of reproducibility ≥ 0.90.

### Appendix A.3. Defeat and Entrapment Scale—Short (DES-S)

The DES-S contains eight items assessing feelings of defeat and perceived inability to escape from adverse circumstances (Griffiths et al., 2015), a core predictor of suicidal ideation. Responses are recorded on a five-point scale (0 = Not at All to 4 = Extremely Like Me). Reported internal consistency across samples ranges from *α* = 0.84 to 0.95.

### Appendix A.4. Mee–Bunney Psychological Pain Assessment Scale (MBPPAS)

The MBPPAS is a 10-item self-report scale assessing psychological pain and distress (Mee et al., 2011). Each item is rated on a five-point Likert scale (0 = Never to 4 = Always). Typical items include “I feel psychological pain greater than any physical pain” and “I feel the only way for psychological pain to stop is to die.” Internal reliability is consistently high (*α* > 0.75).

### Appendix A.5. Childhood Trauma Questionnaire (CTQ)

The CTQ is a 28-item inventory assessing childhood abuse and neglect experiences (Bernstein et al., 2003). Eight items were selected to capture core trauma domains (e.g., “Hit hard enough to leave bruises”). Items are rated on a five-point frequency scale (0 = Never True to 4 = Very Often True). Higher scores indicate greater exposure to early adverse experiences.

### Appendix A.6. Psychological Strain Scales (PSSs)

Twenty items from the PSSs were used to assess perceived incongruence between social expectations and personal values (e.g., “Between chastity and sexual liberty, I don’t know what I should do”). Higher scores reflect greater psychological strain associated with internal conflict and social stress.

### Appendix A.7. Symptom Checklist-90 (SCL-90) Depression Subscale

The depression subscale of the SCL-90 assesses affective, cognitive, and somatic symptoms of depression over the past week. It contains 13 items rated on a five-point scale (0 = Not at All to 4 = Extremely). Typical items include “Feeling low in energy or slowed down,” “Crying easily,” and “Feeling no interest in things.” The scale has demonstrated excellent internal consistency (α = 0.90–0.94) in Chinese university samples. Mean scores are computed, with higher values indicating greater depressive symptomatology.

Note: All scales described above were used as inputs for constructing the CKC composite score. Detailed item wording and scoring procedures for each subscale are presented in Appendix B.

## Appendix B. Item Descriptions and Scoring Procedures

The following items represent the full 45 item pool used in the CKC screening project, from which the 29 most predictive items were algorithmically selected for the final model. To ensure transparency and replicability, all items from the original pool are presented below, even though only 29 were retained in the optimized CKC framework. These items include both explicit indicators (suicidal ideation, personal/family psychiatric history, health and medication history) and implicit psychological indicators (depression, self-esteem, defeat and entrapment, psychological strain, psychological pain, and childhood trauma). All items were rated on 0–4 Likert-type scales unless otherwise specified. The HDSQ suicidal ideation subscale was included as part of explicit indicators but not as a direct self-harm measure, in compliance with ethical guidelines.

**Table A1:** Rosenberg Self-Esteem Scale (RSE).

Item	Scoring
I feel that I’m a person of worth.	4 3 2 1 0
I take a positive attitude toward myself.	4 3 2 1 0
I feel I do not have much to be proud of. (R)	0 1 2 3 4
I feel that I have a number of good qualities.	4 3 2 1 0

*Note.* (R) indicates a reverse-coded item. 0 = Not at All; 1 = A Little Bit; 2 = Moderately; 3 = Quite a Bit; 4 = Extremely Like Me.

**Table A2:** Defeat and Entrapment Scale—Short (DES-S).

Item	Scoring
I feel powerless.	0 1 2 3 4
I feel that I am one of life’s losers.	0 1 2 3 4
I feel that there is no fight left in me.	0 1 2 3 4

*Note*. 0 = Not at All; 1 = A Little Bit; 2 = Moderately; 3 = Quite a Bit; 4 = Extremely Like Me.

**Table A3:** Psychological Strain Scales (PSS).

Item	Scoring
I have the same qualities as my friends, but they are paid much more than I am.	0 1 2 3 4
I wish I could be successful, but there are too many obstacles in my life.	0 1 2 3 4
I think society is unfair to me.	0 1 2 3 4
When I have a problem, I always stay alone and away from others.	0 1 2 3 4
Between chastity and sexual liberty, I don’t know what I should do.	0 1 2 3 4

*Note*. 0 = Not at All; 1 = A Little Bit; 2 = Moderately; 3 = Quite a Bit; 4 = Extremely Like Me.

**Table A4:** Mee–Bunney Psychological Pain Assessment Scale (MBPPAS).

Item	Scoring
In the past month, my psychological pain has exceeded any physical pain.	0 1 2 3 4
I feel like the only way for psychological pain to stop is to die.	0 1 2 3 4

*Note.* 0 = Never; 1 = Rarely; 2 = Half the time; 3 = Often; 4 = All the time.

**Table A5:** Childhood Trauma Questionnaire (CTQ).

Item	Scoring
Was looked out for.	0 1 2 3 4
Some people in my family think I’m worthless.	0 1 2 3 4
Got taken care of.	0 1 2 3 4
Hit hard enough to leave bruises.	0 1 2 3 4
My childhood was unhappy.	0 1 2 3 4
Felt loved. (R)	4 3 2 1 0
Was sexually abused.	0 1 2 3 4

*Note.* (R) indicates a reverse-coded item. 0 = Never True; 1 = Rarely true; 2 = Sometimes true; 3 = Often true; 4 = Very Often True.

**Table A6:** SCL-90 depression subscale.

Item	Scoring
Feeling low in energy or slowed down.	0 1 2 3 4
Crying easily.	0 1 2 3 4
Feeling no interest in things.	0 1 2 3 4
Feeling hopeless about the future.	0 1 2 3 4
Feeling lonely.	0 1 2 3 4
Feeling blue.	0 1 2 3 4
Thoughts of ending your life.	0 1 2 3 4
Feeling trapped or caught.	0 1 2 3 4
Feeling worthless.	0 1 2 3 4
Feeling that everything is an effort.	0 1 2 3 4
Feeling no interest or pleasure in things.	0 1 2 3 4
Feeling that people dislike you.	0 1 2 3 4
Feeling that life is not worthwhile.	0 1 2 3 4

*Note*. 0 = Not at All; 1 = A Little Bit; 2 = Moderately; 3 = Quite a Bit; 4 = Extremely.

**Table A7:** HDSQ suicidal ideation subscale.

Item	Scoring
Item 1: Suicidal thoughts	0–3 graded statements from no thoughts to always having thoughts
Item 2: Planning level	0–3 graded from no plan to definite plan
Item 3: Control over thoughts	0–3 graded from full control to no control
Item 4: Impulses to kill oneself	0–3 graded from none to always

*Note.* Please read each group of statements carefully and select the one that best describes you during the past six months.

**Table A8:** Other risk indicators.

Item	Scoring
Have you ever had suicidal behaviour?	0/1
Do you currently feel that you have health problems?	0/1
Have you ever taken medication for psychological problems?	0/1
Family history of bipolar disorder (parents, siblings, or other relatives)	✓ if applicable
Family history of depression (parents, siblings, or other relatives)	✓ if applicable
Family history of suicide (parents, siblings, or other relatives)	✓ if applicable
Other mental disorders in relatives	✓ if applicable

*Note.* A check mark (✓) indicates that the condition applies. All subscale mean scores were computed as inputs to the CKC model. Higher mean scores indicate higher levels of risk for each construct. 0 = No; 1 = Yes.

## Appendix C

**Table A9:** Performance of machine learning models in suicide risk classification.

	University A					University B				
Method	Sensitivity	Specificity	PPV	NPV	Youden	Sensitivity	Specificity	PPV	NPV	Youden
LR_Normal	0.056	0.999	0.400	0.988	0.055	0.063	0.999	0.111	0.997	0.061
LR_Smote	0.972	0.879	0.092	1.000	0.851	0.875	0.887	0.020	1.000	0.762
RF_Normal	1	1	1	1	1	0	1.000	0	0.997	0.000
RF_Smote	1	1	1	1	1	0.375	0.989	0.085	0.998	0.364
Decisiontree_Normal	0.250	1	1	0.991	0.250	0.063	0.999	0.143	0.997	0.061
Decisiontree_Smote	0.944	0.897	0.104	0.999	0.842	0.688	0.880	0.015	0.999	0.567
SVM_Normal	0	1	NaN	0.988	0	0	1	NaN	0.997	0
SVM_Smote	0.889	0.921	0.124	0.998	0.810	0.750	0.920	0.025	0.999	0.670
KNN_Normal	0.083	1.000	0.750	0.989	0.083	0.063	0.999	0.167	0.997	0.062
KNN_Smote	1	0.960	0.238	1	0.960	0.500	0.916	0.016	0.999	0.416
Bayesian_Normal	0.639	0.888	0.067	0.995	0.527	0.938	0.906	0.026	1.000	0.843
Bayesian_Smote	0.778	0.826	0.053	0.997	0.603	0.938	0.853	0.017	1.000	0.790
MLP_Normal	0.278	0.998	0.667	0.991	0.276	0.125	0.998	0.167	0.998	0.123
MLP_Smote	1	0.995	0.72	1	0.995	0	0.985	0	0.997	−0.015

*Note.* This classification report summarizes the performance of multiple algorithms across key diagnostic metrics, including sensitivity, specificity, positive predictive value (PPV), negative predictive value (NPV), and Youden’s index. Youden’s index is a widely used measure of diagnostic accuracy, defined as (sensitivity + specificity − 1). NaN indicates “Not a Number,” meaning that PPV was undefined because no positive cases were predicted. “SMOTE” denotes models trained on datasets balanced using imblearn’s Synthetic Minority Oversampling Technique, whereas “Normal” indicates models trained on the original unbalanced data. To verify model stability, additional cross-validation was conducted by reversing the training and test sets (i.e., using University B as the training set and University A as the test set). Results remained consistently suboptimal, underscoring the limitations of conventional machine learning methods for low-prevalence outcomes such as suicide risk prediction.

**Table A10:** Sensitivity and specificity of university suicidal ideation—tracked data.

	0 (No Suicide Risk)	1 (Suicide Risk)	Sensitivity	Specificity	Youden	PPV	NPV
count	5892	42					
Risk Score ≥ 8	974	37	0.8810	0.8347	0.7156	0.0366	0.9990
Risk Score ≥ 9	768	37	0.8810	0.8697	0.7506	0.0460	0.9990
Risk Score ≥ 10	651	36	0.8571	0.8895	0.7467	0.0524	0.9989
Risk Score ≥ 11	544	36	0.8571	0.9077	0.7648	0.0621	0.9989
Risk Score ≥ 12	494	35	0.8333	0.9162	0.7495	0.0662	0.9987
Risk Score ≥ 13	472	35	0.8333	0.9199	0.7532	0.0690	0.9987
Risk Score ≥ 14	457	35	0.8333	0.9224	0.7558	0.0711	0.9987
Risk Score ≥ 15	447	35	0.8333	0.9241	0.7575	0.0726	0.9987
